# Microstructural and Mechanical Property Variations in 316L Stainless Steel Fabricated by Laser Powder Bed Fusion Under High-Density Processing Conditions

**DOI:** 10.3390/ma18163899

**Published:** 2025-08-20

**Authors:** Shun Zhang, Xudong Wu, Zhong Wang, Meiling Jiang, Guoliang Huang, Xiaoqiang Peng, Chen Yang, Junyan Zhu, Ke Huang

**Affiliations:** 1 AECC Chengdu Enging Co., Ltd., Chengdu 610500, Chinawangzhongmyname@163.com (Z.W.); hgl_sugar@163.com (G.H.); cyang127@alumni.sjtu.edu.cn (C.Y.); 2 College of Materials Science & Engineering, Sichuan University, Chengdu 610065, China; m17311044998@163.com (X.W.); jml057589@163.com (M.J.); a2966375864@outlook.com (X.P.); 3 Taihang Laboratory, Chengdu 610213, China

**Keywords:** 316L austenitic stainless steel, laser powder bed fusion, mechanical properties, process optimization

## Abstract

It has become a trend to precisely control the additive manufacturing process parameters within the high-density process window to obtain high-performance metal parts. However, there are few reports on this topic currently, leaving this research without sufficient references. This study took 316L austenitic stainless steel as a case study. In total, 36 groups of specimens were manufactured by Laser powder bed melting (LPBF), and then, two highly dense specimens were selected to study the variation in their microstructure and properties. The densities of the selected specimens, S1 (VED = 81 J/mm^3^) and S2 (VED = 156.3 J/mm^3^), are 99.68% and 99.99%, respectively. The results indicated that, compared with the S1 specimen, the S2 specimen significantly decreased in terms of yield strength (YS), ultimate tensile strength (UTS), and elongation (EL), which are 7.28%, 6.34%, and 19.15%, respectively. The differences in mechanical properties were primarily attributed to differences in their microstructures. Further, compared with the S1 specimen, the fitted ellipse aspect ratio and average grain size of the S2 specimen increased by 79.88% and 53.45%, respectively, and the kernel average misorientation (KAM) value and geometric necessary dislocation (GND) density increased by 36.00% and 58.43%, respectively. Furthermore, the S1 specimen exhibited a strong texture in the <101>//Z direction, whereas no obvious texture was observed in the S2 specimen. Obviously, the reason why precise regulation within the dense parameter range can achieve better performance is that the microstructure and mechanical properties of the specimens prepared within the dense range are different. More importantly, this study provides a feasible framework for optimizing alloys with broad and dense parameter ranges, demonstrating the potential to achieve high-performance components through precise parameter control. Furthermore, the results reveal that even within a wide range of high-density forming parameters, significant variations in microstructure and mechanical properties can arise depending on the selected parameter combinations. These findings underscore the critical importance of meticulous process parameter optimization and microstructural regulation in tailoring material properties.

## 1. Introduction

**Figure 1:**
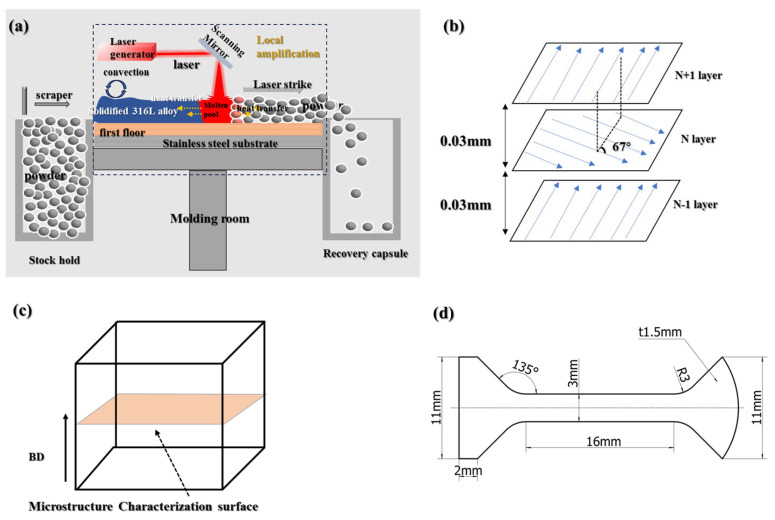
(**a**) Schematic diagram of LPBF principle, (**b**) printing method, (**c**) characterization cross−section, (**d**) tensile specimen model. Reprinted from Ref. [17].

Additive manufacturing (AM) has emerged as a transformative technology in advanced manufacturing, offering unparalleled capabilities in near-net shaping, complex geometry fabrication, and rapid component repair. These attributes significantly enhance design flexibility while reducing material waste and production lead times [1,2,3,4]. AM technologies are broadly categorized into directed energy deposition (DED) and powder bed fusion (PBF), with laser powder bed fusion (LPBF, illustrated in Figure 1a) representing the most industrially mature PBF variant. LPBF enables the production of near-fully dense components (relative density ≥ 99.99% [5]), making it particularly suitable for applications requiring high structural integrity and geometric precision.

In laser powder bed fusion (LPBF), identifying optimal processing parameters—including laser power (P), scanning speed (v), and hatch spacing (h)—is critical for producing high-performance metallic components. Current parameter optimization strategies predominantly prioritize achieving maximal density. For instance, Wu et al. [6] and Zhang et al. [7] defined minimum porosity as the optimization criterion for FeCoNiCrMn high-entropy alloys, while Shen et al. [8] and Banerjee et al. [9] employed machine learning (ML) to iteratively predict relative density for LPBF parameter refinement. However, this density-centric approach becomes suboptimal for alloys exhibiting broad high-density processing windows, such as 316L stainless steel [10,11], titanium alloys [12], Invar alloys [13,14], and Inconel 718 [15]. For such materials, density alone fails to discriminate microstructure–property relationships within the high-density regime. Consequently, microstructure engineering through precise parameter control—beyond mere density optimization—is essential to unlock superior mechanical performance and process reproducibility.

Recent advances in microstructure engineering have demonstrated the critical role of process parameter optimization in tailoring material properties for additive manufacturing [16]. For instance, Huang et al. [17] studied LPBF316L stainless steel and found that when the density exceeded 99.5%, the mechanical properties no longer showed a linear relationship with density. Performance variations in this range were mainly due to microstructural differences. Thus, they stressed that when selecting optimal processing parameters in this high-density range, microstructural features should be considered, not just density. Eom et al. [18] modulated the mechanical performance of LPBF-processed AlSi10Mg alloys by strategically controlling microstructure heterogeneity. Similarly, Yu et al. [19] enhanced the strength–ductility synergy in eutectic high-entropy alloys (EHEAs) through post-process heat treatments that refined phase distributions. In a novel approach, Zhan et al. [20] achieved tunable phase transformation behavior and superelasticity in NiTi alloys via multi-region repeated laser scanning, enabling localized microstructure customization. In addition, alloying elements can significantly refine the microstructure and enhance the mechanical properties of materials. For example, the addition of Cu to LPBF TC4 alloy facilitates the formation of fully equiaxed and fine β grains, thereby improving the strength and toughness of the titanium alloy [21]. Adding TiC to LPBF super duplex stainless steel promotes the formation of micron-sized TiC particles, submicron M23C6 particles, and TiCxNy nanoparticles, along with grain refinement, whose effects work together to improve both strength and toughness [22]. Adding the B element to LPBF 316L stainless steel can form a M_2_B strengthening phase distributed among the cellular structure, thereby increasing its strength by 1.84 times and hardness by 1.67 times [23]. These studies collectively underscore that precise parameter adjustments—spanning energy input, thermal management, and scanning strategies—can profoundly influence grain morphology, phase composition, and defect distribution, thereby dictating macroscopic mechanical responses. Although the performance of alloys can be enhanced through adjustments to process parameters, chemical composition, heat treatment, etc., optimizing process parameters is often the most straightforward approach. Appropriate process parameters are not only essential for alloy forming but also function as an in situ heat treatment method to regulate microstructure and material properties [17,24]. However, the influence of LPBF process parameters on material performance has been predominantly focused on density, with defects generally regarded as the primary factor affecting performance. Fewer studies have addressed the variations in microstructure and mechanical properties within the range of fully dense materials.

To further explore the differences in mechanical properties and microstructure between different parameters in the high-density parameter range and reveal the intrinsic mechanisms affecting its properties, this work selected a widely used commercial 316L stainless steel as an example, and selected two sets in the high-density parameter range for mechanical property testing and microstructure characterization. The mechanical properties, fracture surfaces, and microstructures of the two sets are compared and investigated in detail.

## 2. Materials and Experimental Methods

### 2.1. LPBF Equipment and Raw Materials

**Figure 2:**
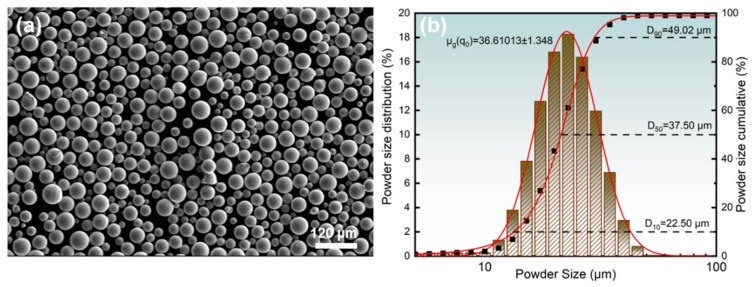
316L stainless steel powder: (**a**) SEM morphology; (**b**) particle size distribution.

The specimens were fabricated using an HBD-80 laser powder bed fusion system (Hambone United 3D Technology Co., Ltd., Shanghai, China), equipped with a YLR-500 fiber laser (wavelength: 1064 nm; beam diameter: ≈33 μm). As illustrated in Figure 1a, the LPBF process employed a bidirectional interlayer rotation strategy with a 67° angular offset between successive layers (Figure 1b) to mitigate directional anisotropy. The 316L stainless steel powder used in this study was gas-atomized (supplied by Beijing Yanbang New Material Technology Co., Ltd., Beijing, China). The chemical composition was obtained through X-ray fluorescence spectrometry analysis (XRF) in Table 1. As shown in Figure 2a, the powder particles exhibit near-spherical morphology, while Figure 2b presents a log-normal size distribution characterized by a median diameter (D_50_) of 37.50 μm. This optimized particle geometry and size distribution ensure excellent flowability and layer-wise packing density, fulfilling the stringent feedstock requirements for laser powder bed fusion (LPBF) processing.

**Figure 3:**
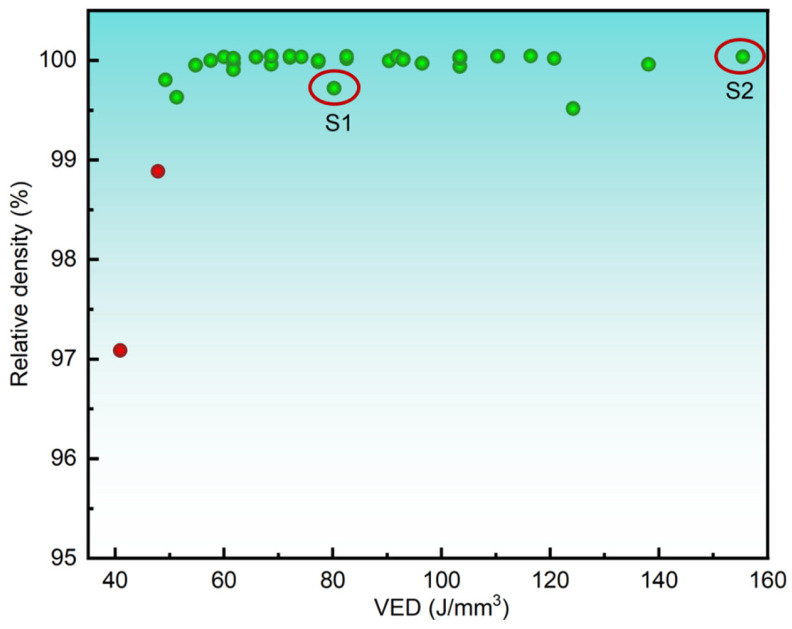
Relationship between relative density and volumetric energy density of LPBF-manufactured 316L stainless steel.

The volumetric energy density (VED = P/(h⋅v⋅t)), where P denotes laser power (W), h is hatch spacing (μm), v represents scanning speed (mm/s), and t is layer thickness (μm), was employed to systematically evaluate parameter effects on 316L stainless steel quality. A full-factorial experimental matrix comprising 36 parameter combinations was designed, spanning P = 150–225 W, v = 600–1000 mm/s, and h = 80–120 μm (layer thickness fixed at t = 30 μm). Figure 3 demonstrates a nonlinear correlation between VED and relative density (RD), revealing a plateau regime (VED > 80 J/mm^3^) where RD exceeds 99.5%. This work analyzed a total of 12 sets of experimental parameters. Based on different energy inputs (VED), two representative VED conditions—81.0 J/mm^3^ (S1) and 156.3 J/mm^3^ (S2)—were selected for comparative analysis to decouple the microstructure–property relationships independently of density variations. Detailed parameters are summarized in Table 2.

### 2.2. Mechanical Property Testing

Tensile testing was performed using a custom-developed high-throughput testing system to evaluate mechanical properties under multiple parameter conditions. This machine is manufactured by Shenzhen Wan Test Equipment Co., Ltd. (Shenzhen, China), with the model number TSE504C and a specification of 50 kN. Specimen geometry followed ASTM E8 subscale standards [27], with a gauge thickness of 1 mm and critical dimensions illustrated in Figure 1d. Triplicate specimens were fabricated for each parameter set to ensure statistical reliability, with mean values calculated from three valid tests. Quasi-static uniaxial loading was applied at a constant crosshead speed of 2 mm/min.

### 2.3. Material Characterization

Landscape printing was adopted, and the microstructure was characterized in the horizontal cross-sectional direction (perpendicular to the BD direction), as shown in Figure 1c. The specimen density was measured by a MAY-D80 automatic test balance using the Archimedean drainage method. The microstructure of the specimen was observed by a Zeiss 40MAT metallographic microscope (ZEISS, Oberkochen, Germany). EBSD images were obtained using the Zeiss Gemini 300 SEM and electron backscattering diffraction (EBSD) technique (ZEISS, Oberkochen, Germany). Electrochemical polishing with a 1:9 volume ratio of perchloric acid and anhydrous ethanol solution was followed by argon ion polishing with Gatan Precision Etching Coating System (PECS) II 685 (Gatan, Inc., Pleasanton, CA, USA). Finally, EBSD was tested. The Aztec Crystal 2.1 software was used to process EBSD data.

## 3. Results

### 3.1. Relative Density and Metallurgical Examination

**Figure 4:**
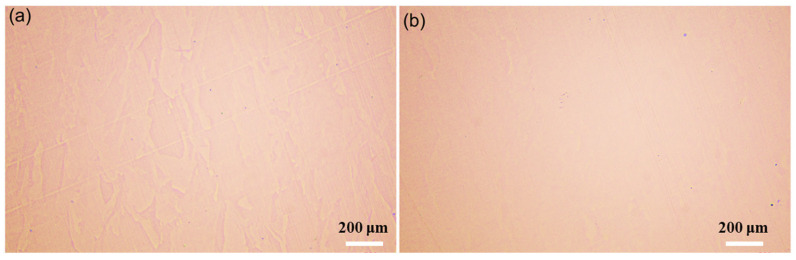
Metallographic micrographs: (**a**) S1 (81.0 J/mm^3^); (**b**) S2 (156.3 J/mm^3^).

The relationship between VED and relative density is shown in Figure 3. Using the Archimedean drainage method to determine the density, it is obvious that the density of most data points is greater than 99.50% (green data points), and only two data points are less dense (red data points) [17]. The relative densities of the S1 and S2 specimens are 99.68% and 99.99%, respectively. At the same time, VED ranges from 50.0 J/mm^3^ to 156.3 J/mm^3^, proving that SS 316L has a wide range of molding parameters. Figure 4 shows metallographic microscopic images of the S1 and S2 experimental groups. It can obviously be observed that the number and size of the pores are small, and the overall density is excellent, which is consistent with the density test results.

### 3.2. XRD Diffraction

**Figure 5:**
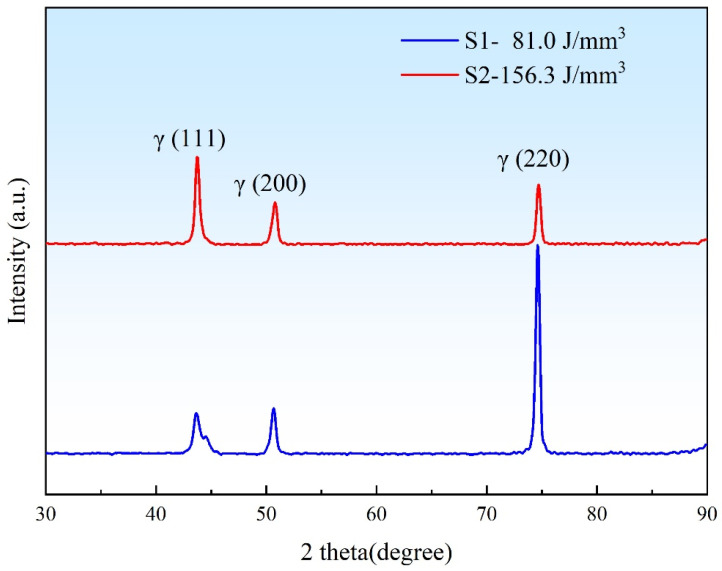
XRD diffraction pattern.

Figure 5 shows the XRD diffraction patterns of the S1 and S2 specimens, which are typical FCC (γ-Fe) diffraction patterns, and no obvious ferrite can be observed. Clearly, the positions of the diffraction peaks in the two sets of experiments are consistent, with the difference lying in the strongest peaks. Among them, the strongest diffraction peak of the S1 specimen is the (220) crystal plane, and the strongest peak of the S2 specimen is the (111) crystal plane. However, the intensities of the three groups of peaks in the S2 specimen are similar. Thus, it can be seen that the S1 specimen has a certain preferred orientation on the (220) crystal plane, while no obvious preferred orientation can be observed in the S2 specimen. Through the analysis of the XRD patterns of the S1 and S2 specimens, it is proven that within the same dense range, the crystal orientation will change with different process parameters, emphasizing the importance of precisely controlling process parameters for material performance. Similar results have also been observed in other studies.

### 3.3. Mechanical Properties and Fracture Morphology

**Figure 6:**
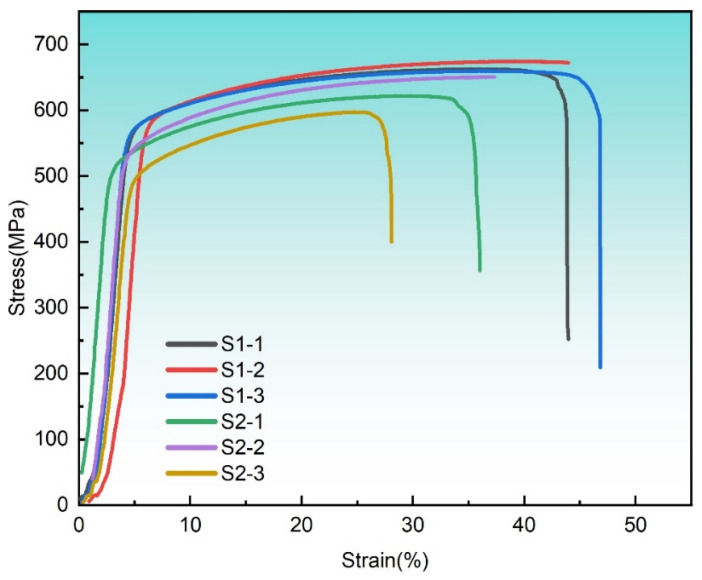
Tensile stress–strain curves of S1 (81.0 J/mm^3^) and S2 (156.3 J/mm^3^).

The strength and ductility of materials are important indicators for evaluating the mechanical properties of materials. The tensile curve obtained through the tensile test in this work is shown in Figure 6. Three groups of repeated specimens were set for S1 and S2, and their average values were obtained to characterize their mechanical properties, including yield strength (YS), ultimate tensile strength (UTS), and elongation (EL). The statistical mean results are shown in Table 3. Figure 6 systematically shows the difference between S1 and S2 in terms of EL, YS, and UTS. It can be seen from the figure that when VED increases from 81.0 J/mm^3^ to 156.3 J/mm^3^, YS, UTS, and EL all decrease to 7.28% (∆YS = 41.21 MPa), 6.34% (∆UTS = 42.18 MPa), and 19.15% (∆EL = 8.84%), respectively. Also, the standard deviations in the YS, UTS, and EL of S2 are higher than those of S1 and are 17.03, 26.73, and 10.15, respectively. This indicates that the S1 process was more stable than the S2 process. It was found that even if the density of the S1 specimen is lower than that of the S2 specimen (S1 = 98.68%; S2 = 99.99%), its mechanical properties are not necessarily superior but will show a downward trend. Therefore, when selecting the best process parameters in the range of dense parameters, further consideration of factors other than relative density is extremely important.

**Figure 7:**
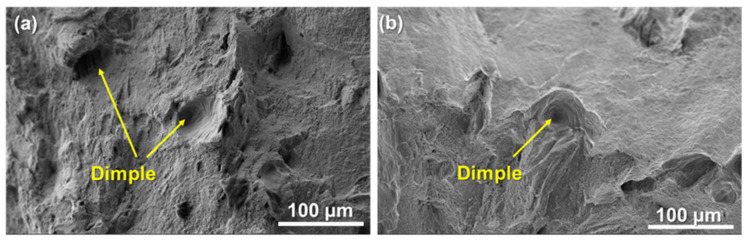
Fracture morphology of tensile specimens photographed by SEM: (**a**) S1; (**b**) S2.

**Figure 8:**
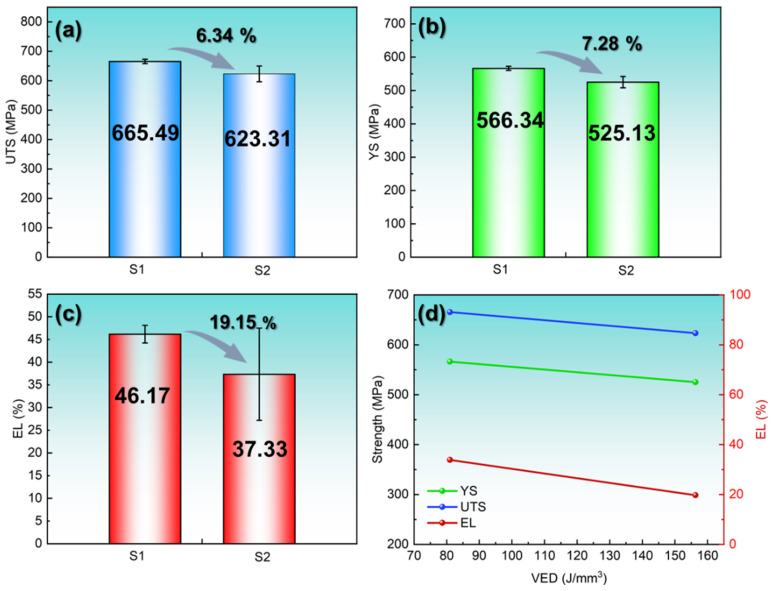
Mechanical property differences between S1 and S2 specimens: (**a**) ultimate tensile strength (UTS), (**b**) yield strength (YS), (**c**) elongation (EL), (**d**) relationship between mechanical properties and VED.

In order to further analyze the fracture surface morphology of the S1 and S2 specimens after testing, scanning electron microscopy (SEM) was utilized to characterize their fracture surface, as shown in Figure 7. In general, the fracture surfaces of the S1 and S2 specimens showed many pits (dimples), and some pits were about 50 μm in size, which is a typical feature of ductile fracture. The existence of the dimple means the material can absorb a large amount of energy, and its size and depth will directly affect the ductility of the material [28]. As VED increased from 81.0 J/mm^3^ to 156.3 J/mm^3^, the number and size of the dimples decreased, and a suspected local brittle fracture was observed in Figure 7b, which also coincides with the decrease in YS, UTS, and EL in the stretching results in Figure 8.

### 3.4. Microstructure

#### 3.4.1. Grain Size and Morphology

**Figure 9:**
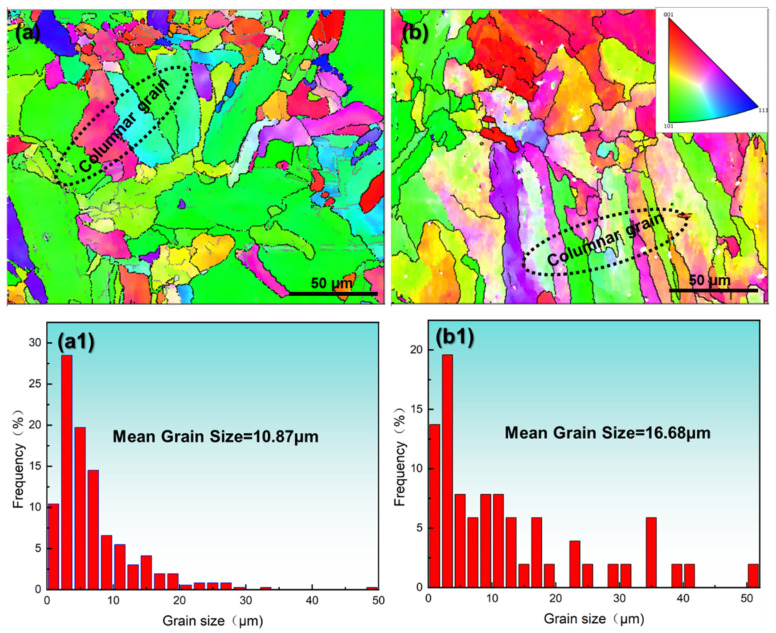
IPF diagram (**a**,**b**) and average grain size distribution diagram (**a1**,**b1**): (**a**,**a1**) S1; (**b**,**b1**) S2.

**Figure 10:**
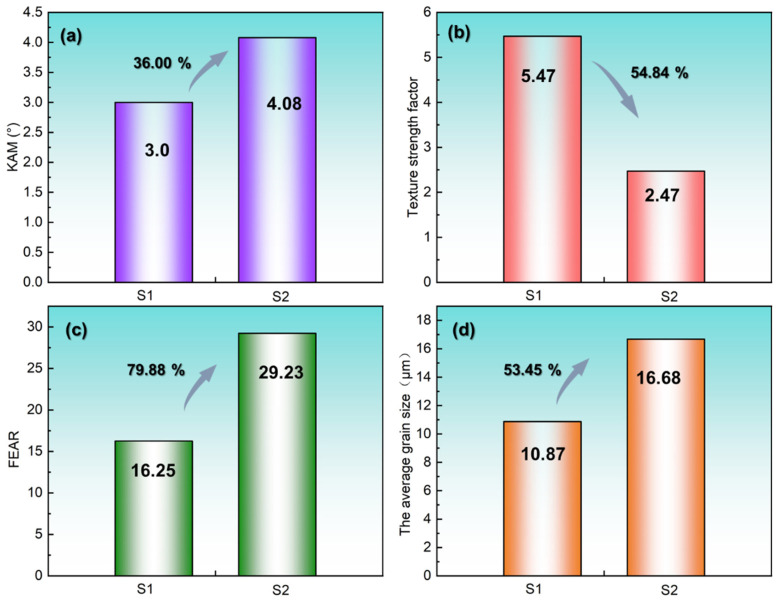
Comparison between the parameters of S1 and S2: (**a**) kernel average misorientation (KAM), (**b**) texture orientation factor, (**c**) FEAR, (**d**) average grain size.

Electron backscatter diffraction (EBSD) analysis of the SS316L specimens (S1 and S2) reveals distinct grain morphology and size distributions under varying laser powder bed fusion (LPBF) parameters, as illustrated in Figure 9 (tested in the XOY plane; see Figure 1c). Both specimens predominantly exhibit columnar grains with limited equiaxed structures. Increasing the volumetric energy density (VED) from 81.0 J/mm^3^ to 156.3 J/mm^3^ induces a rightward shift in the grain size histogram (Figure 9(a1,b1) and Figure 10d), elevating the average grain size from 10.87 μm to 16.68 μm. At the higher VED (156.3 J/mm^3^), maximum grain dimensions exceed 50 μm, accompanied by a pronounced increase in columnar grain fraction. This microstructural coarsening correlates with reduced yield strength (YS), ultimate tensile strength (UTS), and elongation (EL) in tensile tests (Figure 5 and Figure 6), consistent with Hall–Petch relationships. The grain enlargement arises from suppressed nucleation at elevated temperatures, where grain growth outpaces nucleation—a phenomenon documented in Invar alloys [29], titanium alloys [30,31], and steels [32].

Quantitative analysis of the facet ellipse aspect ratio (FEAR) in Figure 10c corroborates this trend: the FEAR increases by 79.88% (S1 = 16.25 vs. S2 = 29.23), confirming enhanced columnar grain dominance at a higher VED. This microstructural transition aligns with classical solidification theory [33,34], wherein the temperature gradient-to-solidification rate ratio (*G*/*R*) governs grain morphology [35]. Elevated VED raises *G*/*R* values [33,36,37], favoring columnar over equiaxed growth. Figure 10 further quantifies differences between S1 and S2 in kernel average misorientation (KAM, +36%), average grain size (+53.45%), and texture strength (+54.84%). These results underscore that parameter optimization within the densification regime critically governs grain morphology, size, and crystallographic texture—a topic expanded on in the subsequent section.

#### 3.4.2. Texture and Dislocation Density

**Figure 11:**
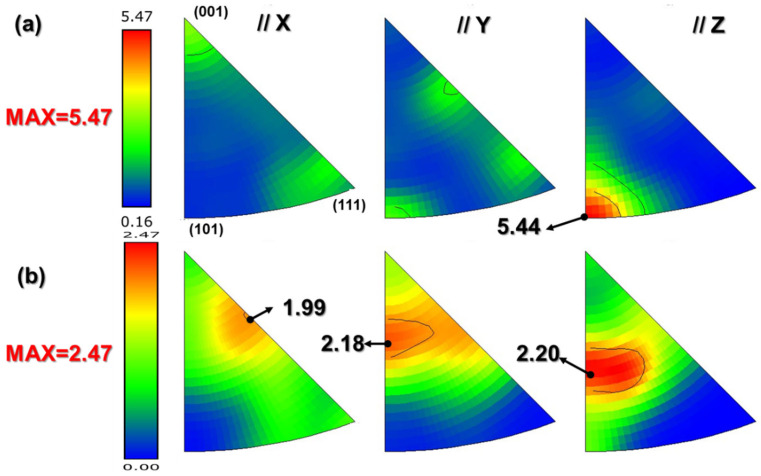
Inverse pole figure: (**a**) S1 (81.0 J/mm^3^); (**b**) S2 (156.3 J/mm^3^).

The inverse polar diagram of SS316L prepared by LPBF technology is shown in Figure 11. According to the reverse polar diagram, the crystal orientation of the grains in the specimen under different parameters can be determined well, and the texture direction can be judged [38]. When VED = 80.0 J/mm^3^, the crystal has a strong preferred orientation at <101>//Z, corresponding to the wider green distribution in Figure 11a. When VED = 156.3 J/mm^3^, the crystal has a certain strength orientation in the X, Y, and Z directions and does not show obvious texture orientation. The disordered color distribution in Figure 11b also illustrates this point. The results show that when VED increases from 80.0 J/mm^3^ to 156.3 J/mm^3^, the crystal texture changes obviously, which is manifested as the crystal texture decreases and the crystal growth orientation increases in the X, Y, and Z directions. Therefore, in the dense interval, the process parameters are highly sensitive to the crystal texture (crystal orientation, number of textures, and texture strength) of the S1 and S2 specimens, which, in turn, have an important influence on the properties of the material. This also means that it is important to consider the effect of parameter changes on the texture in the material when accurately optimizing the parameters in the dense interval.

**Figure 12:**
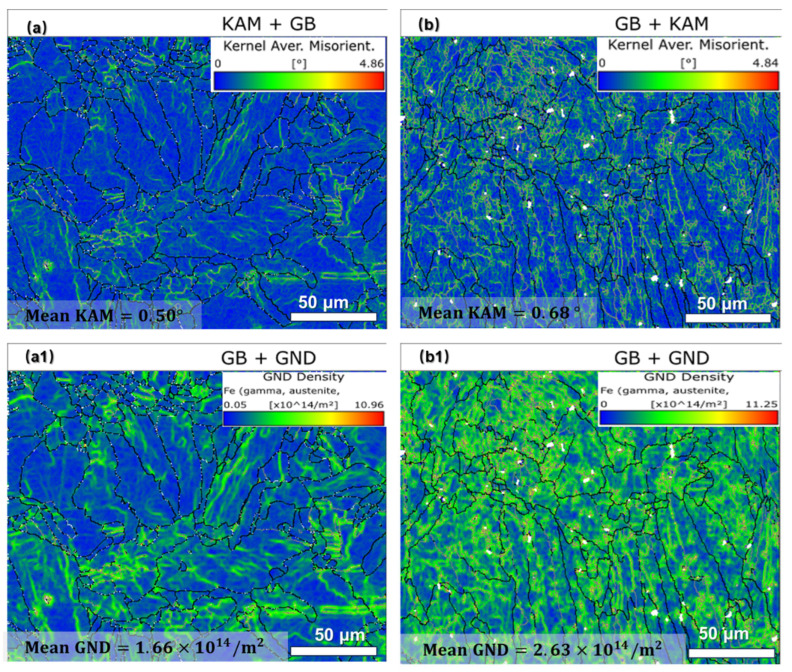
Kernel average misorientation (KAM) (**a**,**b**) and geometric necessary dislocation (GND) (**a1**,**b1**) images: S1 (**a**,**a1**) and S2 (**b**,**b1**).

In laser powder bed fusion (LPBF), the rapid cooling rates and transient thermal gradients inherent to the process lead to significant accumulation of thermal stress within the microstructure. Kernel average misorientation (KAM), a metric derived from electron backscatter diffraction (EBSD), effectively quantifies localized dislocation density and microscopic stress distributions [39]. Similarly, geometrically necessary dislocation (GND) density mapping provides a spatial representation of lattice distortions caused by strain incompatibilities [40]. Figure 12a compares the stress distribution, dislocation density, and KAM values between specimens S1 (Figure 12(a,a1)) and S2 (Figure 12(b,b1)). For S1, the measured KAM and GND values are 0.50° and 1.66 × 10^14^/m^2^, respectively, while S2 exhibits higher values of 0.68° and 2.63 × 10^14^/m^2^. When the volumetric energy density (VED) increases from 81.0 J/mm^3^ to 156.3 J/mm^3^, the KAM and GND values rise by 36.00% and 58.43%, respectively. This trend correlates with microstructural evolution: elevated energy input promotes columnar grain growth and enhances stress dislocation accumulation due to suppressed dynamic recrystallization and intensified thermal shrinkage during solidification. These findings demonstrate that even under high-density conditions, variations in LPBF parameters significantly alter dislocation networks and residual stress states. The observed behavior aligns with classical solidification theory and recrystallization kinetics, wherein higher cooling rates inhibit dislocation annihilation. Consequently, optimizing LPBF parameters requires not only achieving densification but also tailoring microstructural attributes to mitigate stress-induced defects.

## 4. Discussion

### 4.1. The Differences Between the Microstructures

The results demonstrate that within high-density parameter ranges, variations in LPBF processing parameters significantly influence the microstructure and crystallographic characteristics of 316L stainless steel. The key structural parameters evaluated include the mean grain size, kernel average misorientation (KAM), geometrically necessary dislocation (GND) density, grain morphology (quantified by the mean ellipse aspect ratio, FEAR), and texture strength. Notably, when the volumetric energy density (VED) increased from 81.0 J/mm^3^ (S1) to 156.3 J/mm^3^ (S2), distinct differences in grain morphology, dislocation density, and stress distribution were observed. These variations are primarily attributed to the interplay between the rapid solidification dynamics inherent to LPBF and the disparities in laser energy input.

The LPBF process is characterized by rapid solidification rates, wherein an increase in the solidification rate (*R*) corresponds to an exponential reduction in heat input [35]. This phenomenon arises from the localized high heat flux at the melt pool center compared to its boundaries, as dictated by the laser-induced temperature gradient [41]. Under such conditions, the liquid temperature (*T_L_*) remains substantially below the metal’s melting point (*T_M_*), creating significant undercooling (Δ*T*), which promotes heterogeneous nucleation. At higher VED levels, the temperature gradient (*G*) increases while the cooling rate decreases, elevating the *G*/*R* ratio. This shift favors the growth of columnar grains over equiaxed structures.

Residual stress, a critical feature of LPBF-processed components, originates from thermal gradients and solidification-induced shrinkage between adjacent melt pools [42]. Excessive residual stress accumulation can compromise mechanical properties and induce defects [43], as corroborated by the EBSD analysis and mechanical testing results in Section 3.3 (Figure 8). Specifically, the lower VED in S1 generated smaller thermal gradients compared to S2, thereby reducing thermal stresses and dislocation densities. This aligns with the superior mechanical performance observed in S1.

The LPBF process typically induces heterogeneous nucleation at melt pool boundaries, with the epitaxial growth of columnar grains oriented along the maximum temperature gradient. Theoretical models suggest that higher *G*/*R* ratios in S2 enhance undercooling [44,45,46], promoting preferential grain growth along energetically favorable crystallographic directions (e.g., <100> in steels [47,48,49]). However, conflicting reports exist regarding the dominant texture in LPBF-processed 316L, with some studies identifying a <110> texture [50,51,52]. This discrepancy may stem from variations in solidification conditions (e.g., *G* and *R*) that constrain texture evolution [53]. For instance, excessive energy input (as in S2) enlarges the melt pool and reduces cooling rates, fostering competitive growth among grains of differing orientations. Concurrently, intensified Marangoni flows and recoil pressures under high-energy conditions disrupt melt pool stability, further complicating grain growth patterns [54].

In this study, S2 exhibited a steeper thermal gradient than S1 but a slower cooling rate, amplifying orientation-dependent growth competition. The stronger laser-induced thermal shock in S2 also exacerbated grain orientation heterogeneity. These factors collectively explain the diminished texture strength in S2 (156.3 J/mm^3^) relative to S1 (81.0 J/mm^3^).

### 4.2. The Differences Between Mechanical Properties

It is well established that LPBF-manufactured stainless steel exhibits superior yield strength (YS) and ultimate tensile strength (UTS) compared to conventionally processed counterparts [55]. This enhancement is primarily attributed to refined microstructures and dislocation substructures, as described by classical strengthening mechanisms such as the Hall–Petch relationship and Bailey–Hirsch theory [56,57]. While some studies emphasize traditional strengthening contributions—including solid solution, grain boundary, dislocation, and particle strengthening [58,59]—others highlight the role of cellular structures [60]. In this work, we systematically evaluate the effects of dislocations, grain boundaries, and crystallographic texture (Figure 9, Figure 10, Figure 11 and Figure 12) on mechanical performance.

EBSD analyses (Section 3) reveal distinct differences between S1 (VED = 81.0 J/mm^3^) and S2 (VED = 156.3 J/mm^3^). Specifically, S1 demonstrates a 58.3% higher geometrically necessary dislocation (GND) density, a 53.45% smaller average grain size, and a 54.84% stronger texture compared to S2. Correspondingly, S2 exhibits reduced mechanical properties, with decreases of 7.28% in YS, 6.34% in UTS, and 19.15% in elongation (EL) relative to S1. These discrepancies stem from VED-induced microstructural variations, which directly govern mechanical behavior.

The strength of LPBF-processed 316L stainless steel can be modeled as [61]

(1) $$\sigma y=\sigma_{0}+kd^{-0.5}+M\alpha Gb\sqrt{\rho }$$

where G is the shear modulus (84.38 GPa), b is the Burgers vector (2.55 × 10^−10^ m), M is the Taylor factor (2.90), α is a dimensionless constant (0.2–0.3), σ_0_ is the lattice friction stress (183.3 MPa), k is the Hall–Petch coefficient, d is the average grain size, and ρ is the dislocation density [61,62,63,64]. Dislocation density dominates strength contributions, while increased columnar grain fraction and texture intensity reduce mechanical performance.

Grain boundary strengthening (σ_GB_) is quantified as [61]

(2) $$\begin{array}{c} \sigma_{GB}=kd^{-0.5} \end{array}$$

Using k = 0.761 MPa⋅m^−1/2^ [62], the calculated σ_GB_ values for S1 (d = 10.87 μm) and S2 (d = 16.68 μm) are 230.81 MPa and 186.33 MPa, respectively (Figure 9(a1,b1)).

Dislocation strengthening (σ_GND_) follows [61]

(3) $$\begin{array}{c} \sigma_{GND}\;=\;M\alpha Gb\sqrt{\rho } \end{array}$$

With ρ_S1_ = 1.66 × 10^14^ m^−2^ and ρ_S2_ = 2.63 × 10^14^ m^−2^ (Figure 11 and Figure 12), the corresponding σ_GND_ values are 160.79 MPa (S1) and 202.39 MPa (S2).

**Figure 13:**
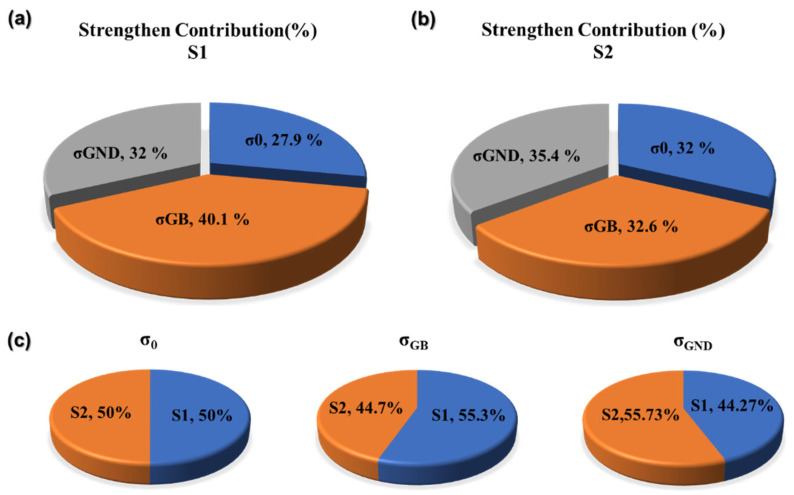
Intensity calculation values: (**a**) S1 specimen, (**b**) S2 specimen, (**c**) comparison of intensity distribution between S1 and S2.

Figure 13 shows the contribution values of each strengthening mechanism to the S1 and S2 specimens, as well as a comparative analysis of the differences between the S1 and S2 specimens under different strengthening mechanisms. Table 4 statistically presents the contribution values of different strengthening mechanisms of specimens S1 and S2 to the yield strength (YS). It is obvious that the grain boundary strengthening of the S1 specimen contributes the most to the yield strength, reaching 230.81 MPa, while in the S2 specimen, the dislocation strengthening contributes the most, reaching 202.39 MPa. Such differences are mainly attributed to the organizational differences caused by the differences in VED. The larger the VED, the more severe the grain coarsening, and the grain boundary strengthening effect decreases. However, at the same time, the dislocation density in the grains increases, and the dislocation strengthening effect gradually increases. Comprehensive analysis shows that the sum of grain boundary strengthening and dislocation strengthening in the S1 specimen is greater than that in the S2. This is consistent with the YS value obtained from the actual test, but the difference in the calculated values is relatively small. The combined strengthening contributions (σ_GB_ + σ_GND_) total 391.6 MPa for S1 and 388.72 MPa for S2. Crucially, S1 derives greater strength from grain boundary refinement, whereas S2 relies more heavily on dislocation hardening. The diminished synergy of these mechanisms in S2—coupled with its coarser microstructure and weaker texture—explains its inferior YS compared to S1.

## 5. Conclusions

To reveal the intrinsic factors affecting the mechanical properties of LPBFed 316L stainless steel with similar density, this work focuses on investigating the mechanical properties and microstructure of 316L stainless steel prepared using two sets of process parameters within high densification parameter intervals. The key conclusions are as follows:

Differences in microstructures within a high-density parameter window. Compared with S1, the average grain size, KAM, geometric dislocation density, and fitted ellipse aspect ratio (FEAR) increased for the S2 specimen, which were 53.45%, 36.00%, 58.43%, and 79.88%, respectively. The proportion of columnar crystals increased. These contributed to the variations in the mechanical properties between S1 and S2.

Differences in mechanical properties within a high-density parameter window. Ultimate tensile strength (UTS), yield strength (YS), and elongation (EL) decreased by 6.34% (UTS = 42.18 MPa), 7.28% (YS = 41.21 MPa), and 19.15% (EL= 8.84%)when VED increased from 81.0 J/mm^3^ (S1) to 156.3 J/mm^3^ (S2). Both the S1 and S2 specimens show obvious ductile fracture characteristics (dimples), but the number and size of dimples in the S2 tensile specimen are smaller, and local brittle fracture phenomena can be found.

Strengthening mechanism. The results indicated that the grain size of the S1 specimen is smaller, and the dislocation density is lower. The contribution of grain boundary strengthening to its strength reaches 230.81 MPa, and its contribution to the S2 specimen reaches 186.33 MPa. In dislocation strengthening, the dislocation density of the S2 specimen is higher, and the strengthening effect is greater, reaching 202.39 MPa. The comprehensive enhanced contribution (σ_GB_ + σ_GND_) to S1 is 391.6 MPa and to S2 is 388.72 MPa.

This study further illustrates that for metals with a broad range of high-density forming parameters, significant variations in microstructure and mechanical properties can arise between different parameter sets. It underscores the critical need for precise control over both the process parameters and the material’s microstructure to optimize the resulting properties.

## Figures and Tables

**Table 1:** Weight percentage composition (wt.%) of SS 316L powder elements. Reprinted from Ref. [17].

Elements	Fe	Cr	Ni	Mo	Si	Mn	P	S	C	N
ASTM A240 [25]	Bal.	16–18	10–14	2–3	0.50	2	0.045	0.03	0.03	0.1
SS316L [26]	Bal.	17.12	11.01	2.54	0.31	0.66	0.005	0.006	0.021	0.002

**Table 2:** The process parameters of S1 (81.0 J/mm^3^) and S2 (156.3 J/mm^3^).

No.	P (W)	V (mm/s)	h (μm)	t (mm)	VED (J/mm^3^)
S1	175	600	120	0.03	81.0
S2	225	600	80	0.03	156.3

**Table 3:** YS, UTS, and EL of S1 (81.0 J/mm^3^) and S2 (156.3 J/mm^3^).

No.	YS (MPa)	UTS (MPa)	EL (%)
S1	566.34 ± 6.21	665.49 ± 7.63	46.17 ± 1.95
S2	525.13 ± 17.03	623.31 ± 26.73	37.33 ± 10.15

**Table 4:** Contribution values of different strengthening mechanisms to yield strength.

Yield Strength (YS, MPa)	Reinforced Contribution			Calculated Value	Measured Value	Difference Radio (%)
	$\sigma_{0}$	$\sigma_{GND}$	$\sigma_{GB}$			
S1	183.30	160.79	230.81	574.90	566.34 ± 6.21	1.51
S2	183.30	202.39	186.33	572.02	525.13 ± 17.03	8.93

## Data Availability

The original contributions presented in this study are included in the article. Further inquiries can be directed to the corresponding author.
